# Supplementary material for: Reconciling Mining with the Conservation of Cave Biodiversity: A Quantitative Baseline to Help Establish Conservation Priorities
Source: PLoS One. 2016 Dec 20;11(12):e0168348. doi: 10.1371/journal.pone.0168348 (PMC5173368; doi:10.1371/journal.pone.0168348)
Supplement: S1 Dataset — (ZIP) [file pone.0168348.s002.zip › Taxa/Serra Norte/SN_2013/N5S/Lista N5S-28.pdf]

CAVIDADE N5S-0028

Classe	Ordem	Fam/Outros	Gên/Outros	Espécie	S-U	U-S	Zon.
Arachnida	Acari	Lohmanniidae		sp.1		X	P
Arachnida	Amblypygi	Phrynidae	*Heterophrynus*	*longicornis*	1		P
Arachnida	Araneae	Araneidae		jovem	X		E
Arachnida	Araneae	Mysmenidae	*Microdipoena*	sp.1		X	E
Arachnida	Araneae	Ochyroceratidae		jovem	X		E
Arachnida	Araneae	Pholcidae	*Leptopholcus*	sp.1	X	X	E
Arachnida	Araneae	Theridiidae	*Achaearanea*	sp.1		X	E
Arachnida	Araneae	Theridiosomatidae	*Plato*	sp.1	X	X	E/P
Diplopoda	Polydesmida	Chelodesmidae		sp.1	1		E
Entognatha	Collembola	Paronellidae		sp.1	X		E
Entognatha	Collembola	Paronellidae		sp.4	X	X	E/P
Entognatha	Diplura	Campodeidae		sp.1	X		E
Insecta	Blattodea			jovem	1	2	E/P
Insecta	Coleoptera	Dytiscidae		sp.2	X		E
Insecta	Coleoptera	Dytiscidae		sp.B	X		E
Insecta	Diptera	Cecidomyiidae	Cecidomyiinae	sp.	X	X	E
Insecta	Diptera	Ceratopogonidae		jovem	X		E
Insecta	Diptera	Psychodidae	*Pintomyia*	*gruta*		X	P
Insecta	Diptera	Psychodidae	*Sciopemyia*	*sordellii*	X		E
Insecta	Homoptera	Cixiidae		sp.1	X	X	E/P
Insecta	Hymenoptera	Formicidae	*Hypoponera*	sp.1	X		E
Insecta	Isoptera	Termitidae	*Nasutitermes*	sp.		X	E
Insecta	Lepidoptera	Gelechioidea		sp.1		X	E
Insecta	Lepidoptera	Noctuoidea		sp.1		1	E
Insecta	Orthoptera	Phalangopsidae	*Paraclodes*	sp.	3		E
Insecta	Psocoptera	Epipsocidae	*Epipsocus*	sp.1	X		E
Insecta	Psocoptera	Psyllipsocidae		jovem		X	P
Mammalia	Chiroptera	Emballonuridae	*Peropteryx*	sp.		3	P
